# Altered Serum Concentrations of Fat-Soluble Vitamins in Dogs with Inflammatory Protein-Losing Enteropathy

**DOI:** 10.3390/ani16121784

**Published:** 2026-06-09

**Authors:** Federica Cagnasso, Veronica Marchetti, Riccardo Ferriani, Elena Benvenuti, Franca Borella, Enrico Bottero, Francesco Bartoli, Barbara Bruno, Renato Zanatta, Verena Habermaass, Antonio Borrelli, Paola Gianella

**Affiliations:** 1 Department of Veterinary Sciences, University of Turin, 10095 Grugliasco, Italy; federica.cagnasso@unito.it (F.C.); franca.borella@unito.it (F.B.); barbara.bruno@unito.it (B.B.); renato.zanatta@unito.it (R.Z.); antonio.borrelli@unito.it (A.B.); 2 Department of Veterinary Sciences, University of Pisa, 56124 Pisa, Italy; veronica.marchetti@unipi.it (V.M.); verena.habermaass@gmail.com (V.H.); 3 Associazione Professionale Endovet Group, 56126 Roma, Italy; riccardoferriani.rf@gmail.com (R.F.); elena@endovet.it (E.B.); botvet@libero.it (E.B.); 4 Department of Translational Research and New Technologies in Medicine and Surgery, University of Pisa, 56126 Pisa, Italy; francesco.bartoli@med.unipi.it

**Keywords:** canine, chronic enteropathy, diarrhea, IBD, vitamin A, vitamin D, vitamin E

## Abstract

Alterations in fat-soluble vitamins are well documented in humans with inflammatory bowel disease; however, data in dogs with chronic enteropathy and inflammatory protein-losing enteropathy (iPLE) are limited. This prospective case–control study compared serum concentrations of vitamins A, D, and E metabolites in dogs with iPLE and healthy controls and evaluated associations with selected clinicopathological variables at diagnosis (T0) and after one month of therapy (T1). Fifty-eight dogs with iPLE and 50 healthy dogs were enrolled. Twenty dogs with iPLE were available for evaluation at T1. iPLE was defined by chronic gastrointestinal signs, hypoalbuminemia of gastrointestinal origin, and histologic evidence of benign intestinal inflammation with or without lymphangiectasia. Serum retinol, 25-hydroxyvitamin D, and α-tocopherol concentrations were measured by HPLC at T0 and T1. At T0, all vitamin concentrations were significantly lower in iPLE dogs (*p* < 0.0001). A weak positive correlation was found between retinol and α-tocopherol concentrations. No differences were observed according to treatment, diet, or histopathologic findings. At T1, retinol concentrations increased (*p* = 0.01), whereas vitamin D and E metabolite concentrations did not. Dogs fed homemade low-fat diets had lower vitamin D concentrations. These findings indicate decreased serum concentrations of multiple fat-soluble vitamins in dogs with iPLE, suggesting altered fat-soluble vitamin homeostasis, with only partial recovery after treatment.

## Simple Summary

Dogs with chronic intestinal diseases often lose important nutrients, but little is known about how this affects fat-soluble vitamins. This study investigated whether dogs with severe intestinal inflammation causing protein loss have lower levels of vitamins A, D, and E compared to healthy dogs, and whether these levels change after treatment. Fifty-eight affected dogs and fifty healthy dogs were included. At the time of diagnosis, dogs with the disease had significantly lower levels of all three vitamins. Lower vitamin D levels were mildly associated with more severe illness and lower serum albumin concentrations. After one month of treatment, the dogs showed clinical improvement and increased blood protein levels. Vitamin A concentrations increased, whereas vitamin D and E concentrations did not show significant improvement. Dogs fed homemade low-fat diets had lower vitamin D concentrations than those fed highly digestible commercial diets. These findings show that dogs with this intestinal disease commonly have decreased serum concentrations of multiple fat-soluble vitamins, not just vitamin D. Monitoring fat-soluble vitamin levels and selecting appropriate diets may help improve the management of this condition and support better health outcomes in affected dogs.

## 1. Introduction

Inflammatory protein-losing enteropathy (iPLE) in dogs is a condition characterized by chronic gastrointestinal inflammation that leads to excessive loss of plasma proteins, particularly albumin, across the intestinal mucosa into the lumen [1]. Up to 75% of dogs with iPLE may concurrently exhibit intestinal lymphangiectasia (IL). IL may occur as a primary idiopathic disorder, which is rare in dogs, or as a secondary consequence of chronic intestinal inflammation; however, the two forms cannot currently be distinguished using histopathology or other available diagnostic tests. Lymph is rich in proteins and lipids, including long-chain fatty acids, fat-soluble vitamins, chylomicrons, immunoglobulins, macrophages, and cytokines. Dilation and rupture of intestinal lymphatic vessels result in leakage of lymph into the intestinal lumen, leading to the loss of both proteins and lipids. This pathogenetic mechanism may contribute to lipid dysmetabolism in dogs affected by iPLE [1].

Fat-soluble vitamins are essential for mammalian health, and their deficiency has been associated with a wide range of pathological conditions in humans, including neurological, visual, hepatic, hemorrhagic, skeletal, and growth disorders. Vitamin A exerts its biological effects primarily through retinoic acid, which regulates cellular differentiation, lipid metabolism, immune responses, and the maintenance of epithelial barrier integrity [2,3,4,5]. Vitamin E functions as a major antioxidant, modulating lipid membrane oxidation, inflammatory pathways, and immune function [6,7]. Vitamin D plays a key role in calcium homeostasis and also participates in immune and inflammatory regulation [8]. Deficiencies of both water- and fat-soluble vitamins are frequently reported in humans with inflammatory bowel disease (IBD), and their supplementation may therefore represent a useful adjunctive therapeutic strategy [9,10]. Indeed, untreated malabsorption of fat-soluble vitamins can lead to clinical manifestations such as retinal degeneration, liver dysfunction, coagulopathies, and skeletal disorders [11]. A recent comprehensive meta-analysis demonstrated significantly lower circulating concentrations of vitamins A, D, E, and K in patients with Crohn’s disease compared with healthy controls. Moreover, an increased prevalence of hypovitaminosis A and E has been documented in both Crohn’s disease and ulcerative colitis, with disease severity correlating with the frequency of vitamin A and E deficiencies in patients with Crohn’s disease [10,12]. The intestinal absorption of fat-soluble vitamins from the diet depends on the detergent action of bile acids, which facilitate the emulsification of dietary lipids and the formation of mixed micelles that solubilize hydrophobic compounds such as vitamins A, D, E, and K and deliver them to the mucosal surface for uptake by enterocytes. In patients with enteropathies, bile acid malabsorption disrupts micelle formation and impairs the intestinal absorption of fat-soluble vitamins, potentially leading to clinically significant deficiencies [13,14]. Because bile acids are absorbed mainly in the terminal ileum and, to a lesser extent, in the colon, dogs with severe chronic enteritis involving these intestinal segments may be particularly predisposed to deficiencies of fat-soluble vitamins as a consequence of bile acid and lipid malabsorption, as has been reported in humans with Crohn’s disease [15]. However, supplementation with fat-soluble vitamins in deficient patients remains a matter of debate in human medicine, as inappropriate or excessive supplementation has been associated with potential adverse effects [9,12,16].

Few studies have evaluated circulating concentrations of vitamins A, D, and E in dogs. Hypovitaminosis D has been reported in dogs with severe chronic enteropathy (CE), exocrine pancreatic insufficiency, and a range of hepatopathies, including congenital biliary atresia, gallbladder mucocele, hepatic neoplasia, cirrhosis, and extrahepatic portosystemic shunts [17,18,19]. Decreased vitamin A concentrations have also been described in dogs with chronic liver disease and congenital extrahepatic portosystemic shunts [18,19,20]. Alterations in fat-soluble vitamin status have also been increasingly recognized in dogs with CE and iPLE. Among these, vitamin D has been the most extensively studied, with multiple reports describing decreased serum 25-hydroxyvitamin D concentrations in dogs with CE and iPLE, as well as associations with disease severity, inflammatory markers, and clinical outcome [21,22,23,24]. In contrast, data on vitamins A and E in canine CE and iPLE are limited [25]. Available evidence suggests that concentrations of these vitamins may be dysregulated rather than uniformly decreased, likely reflecting complex interactions among intestinal malabsorption, lipid dysmetabolism, inflammation, and hepatic handling [25].

Overall, the current literature reveals a substantial knowledge gap regarding the behavior and clinical relevance of fat-soluble vitamins beyond vitamin D in dogs with iPLE. Therefore, the aims of this study were (1) to compare serum concentrations of vitamin A, D, and E metabolites between dogs with iPLE and healthy control dogs; and (2) to explore their associations with clinicopathological findings both at diagnosis and after one month of therapy.

## 2. Materials and Methods

### 2.1. Study Design and Ethics Approval

This was a prospective case–control study of client-owned dogs with iPLE and healthy dogs. All affected dogs were referred for specialist gastroenterology consultations to the Veterinary Teaching Hospitals of the Universities of Pisa and Turin, as well as to selected referral clinics in north-central Italy, between January 2021 and March 2022. The experimental protocol was approved by the Ethics and Animal Welfare Committee of the University of Turin (protocol number 42, 8 January 2021). All analyses were performed on leftover samples.

### 2.2. Cases and Control Dogs

A total of fifty-eight client-owned dogs diagnosed with iPLE and fifty healthy blood-donor dogs were included in the study. Serum concentrations of vitamin A, D, and E metabolites were subsequently measured in leftover serum samples collected from dogs with iPLE at diagnosis (T0) and after one month of therapy (T1), as well as from healthy blood-donor dogs. In addition, clinical, biochemical, and histological results obtained for diagnostic purposes were recorded. Part of the study population overlapped with that of a recently published study by the authors [26]; therefore, the same inclusion criteria were applied. Briefly, iPLE was defined by the presence of chronic gastrointestinal signs persisting for more than three weeks, hypoalbuminemia of gastrointestinal origin (≤2.8 g/dL), and histological evidence of benign gastrointestinal inflammation, with or without intestinal lymphangiectasia, based on multiple endoscopic biopsies evaluated according to the histopathological standards of the World Small Animal Veterinary Association (WSAVA) Gastrointestinal Standardization Group [27]. For all enrolled dogs, a comprehensive diagnostic workup was performed to exclude infectious, parasitic, hepatic, pancreatic, and other intestinal or extraintestinal diseases. This included a Giardia antigen test, fecal flotation performed on pooled three-day fecal samples, complete blood count, serum biochemistry, urinalysis including urinary protein-to-creatinine ratio, and measurement of serum cobalamin, folate, trypsin-like immunoreactivity (TLI), canine-specific pancreatic lipase (cPL), pre- and post-prandial bile acids, and basal cortisol concentrations. An ACTH stimulation test was performed when basal cortisol concentrations were <2 µg/dL. In addition, all dogs underwent abdominal ultrasonographic examination. Dogs with incomplete diagnostic evaluations, evidence of intestinal neoplasia, or response to dietary management and gut microbiota modulation were excluded. Dogs that had received fat-soluble vitamin supplementation prior to enrollment or between T0 and T1 were also excluded. Gastroduodenoscopy was performed in all dogs, while colonoscopy with ileal intubation was carried out when feasible. Therefore, ileal biopsy samples were available only in a subset of dogs, and the diagnosis of iPLE relied primarily on duodenal histopathology. Endoscopic biopsy specimens were fixed in 10% neutral buffered formalin and submitted for histopathological examination. The nature and severity of structural and inflammatory duodenal lesions were evaluated using a four-point grading system (0 = normal, 1 = mild, 2 = moderate, 3 = severe) [27]. Intestinal lymphangiectasia was defined as lacteal dilation exceeding 25% of the width of the villous lamina propria and was further classified as mild, moderate, or severe, according to WSAVA guidelines. Based on histopathological findings, dogs were further assigned to one of two groups: group 1 included dogs with intestinal inflammation and absent or mild lacteal dilation, whereas group 2 comprised dogs with moderate to severe inflammation associated with moderate to severe lacteal dilation [27]. At T0, signalment, the chronic canine enteropathy clinical activity index (CCECAI), type of ongoing diet (i.e., the diet consumed at the time of study inclusion), and use of glucocorticoids prior to referral were recorded. One month after histopathological diagnosis and initiation of therapy (T1), information regarding dietary and therapeutic interventions between T0 and T1 was collected.

The control group consisted of blood donor dogs and dogs owned by the staff of the Veterinary Teaching Hospitals. Blood donors were selected according to the Italian Ministry of Health guidelines [28]. Dogs were considered healthy based on an unremarkable medical history and physical examination, normal hematobiochemical profile, urinalysis (including urinary protein-to-creatinine ratio), and fecal parasitological examination. In addition, no history of drug administration was reported during the six months prior to enrollment, except for antiparasitic prophylaxis.

### 2.3. Fat-Soluble Vitamins Analysis

Blood samples were collected after a 12 h fasting period into plain plastic tubes (without additives) and allowed to clot before centrifugation. Serum samples were refrigerated shortly after processing, and leftover serum aliquots were stored at −80 °C within a few days of collection. No differences in sample storage conditions were present between healthy dogs and dogs with iPLE. Before analysis, frozen serum samples were thawed at room temperature on a benchtop. Samples stored for more than 24 months were precautionarily excluded from the analyses [29,30]. Standard stock solutions were stored at −80 °C and were stable for the duration of the study. Pre-analytical sample handling included protection from light during all processing steps to minimize photodegradation of retinol and α-tocopherol. Concentrations of 25-hydroxyvitamin D, α-tocopherol, and retinol were measured in batch using high-performance liquid chromatography (HPLC-DAD; JASCO Europe, Cremella, Italy) and expressed as nmol/mL. Chromatographic conditions, sample preparation procedures, and analytical validation followed the protocol previously described by Habermaass et al. in 2025 [18]. Calibration curves were prepared from stock standard solutions of 150 nmol/mL for retinol and α-tocopherol and 150 ng/mL (0.377 nmol/mL) for 25-hydroxyvitamin D (Merck, Milan, Italy). Working calibration standards were obtained by serial dilution of the stock solutions in methanol and covered the following ranges: 0 to 100 nmol/mL for retinol and α-tocopherol, and 0 to 0.3774 nmol/mL for 25-hydroxyvitamin D. The lowest calibration level represented the lower end of the working range and was not separately validated as a lower limit of quantification (LLOQ). Concentrations below the lowest calibration standard were estimated by extrapolation from the calibration curve. Hexane, ethanol, and methanol were purchased from the same supplier (Merck, Milan, Italy). Diode-array detector (DAD) wavelengths were set at 280 nm for 25(OH)D, 325 nm for retinol, and 292 nm for α-tocopherol, and analytes were separated using a Luna LC18 column (150 × 2.6 mm I.D., 5 µm particle size) maintained at 25 °C. The chromatographic run time was 20 min. For sample preparation, an aliquot of 200 µL of serum was transferred into a 10 × 1.2 cm glass tube, and 200 µL of ethanol was added. After vortexing for 10 s, the solution was extracted twice with 1.5 mL of hexane containing 12.5 mg/L butylated hydroxytoluene as an antioxidant. After a second vortexing for 60 s, samples were centrifuged at 15,000× *g* for 5 min at 4 °C. The organic phase was collected and evaporated under a nitrogen stream. The entire extraction procedure was performed in a dark room, with the evaporator wrapped in aluminum foil to further shield the samples from light. The dry residue was reconstituted in 200 µL of methanol, and 100 µL was injected into the HPLC system. The mobile phase consisted of 95% methanol delivered at a flow rate of 0.6 mL/min.

### 2.4. Statistical Analysis

All statistical analyses were performed using commercially available software (GraphPad Prism version 9.5.1; Dotmatics, San Diego, CA, USA). All variables were tested for normality using the Shapiro–Wilk test. Student’s *t*-test was used to compare normally distributed variables, whereas the Mann–Whitney U test was used for non-normally distributed variables. Comparisons of paired variables were performed using a paired *t*-test or the Wilcoxon matched-pairs signed-rank test, depending on data distribution. Pearson’s or Spearman’s correlation coefficients were used to assess correlations between variables, as appropriate. Specifically, correlations were explored between fat-soluble vitamin concentrations and selected variables, including age, CCECAI, and serum total protein, albumin, and cholesterol concentrations. All results are reported as median and range (minimum–maximum). Statistical significance was set at *p* < 0.05.

## 3. Results

### 3.1. Population

Among dogs with iPLE, 23 were female (15 spayed) and 35 were male (2 neutered). In the healthy control group, 29 dogs were female (12 spayed) and 21 were male (6 neutered). Sex distribution did not differ significantly between the two groups. Among dogs with iPLE, 10 were mixed-breed (17.2%) and 48 were purebred (82.8%), represented as follows: German Shepherd (*n* = 9); English Setter (*n* = 4); Labrador Retriever, Golden Retriever, and Maltese Dog (*n* = 3 each); Australian Shepherd, Border Collie, Bracco Italiano, Dachshund, and Spanish Breton (*n* = 2 each); Boston Terrier, Bolognese Dog, Bernese Mountain Dog, Belgian Shepherd, Cesky Terrier, Cocker Spaniel, Czechoslovakian Wolfdog, Jack Russell Terrier, Miniature Schnauzer, Pinscher, Pitbull, Podenco, Pug, Spanish Levriero, Springer Spaniel, and Yorkshire Terrier (*n* = 1 each). Among healthy dogs, 16 (32.0%) were mixed-breed and 34 (68.0%) were purebred, represented as follows: Labrador Retriever (*n* = 5); French Bulldog (*n* = 3); Golden Retriever, Jack Russel Terrier, and Galgo (*n* = 2 each); Breton, Maremma Sheepdog, Newfoundland, Pinscher, Dachshund, Dobermann, Bracco Italiano, Shiba Inu, American Staffordshire Terrier, Rottweiler, German Shepherd, Cavalier King Charles Spaniel, West Highland White Terrier, Pekingese, Basset Hound, Cocker Spaniel, Greyhound, Australian Shepherd, Pit Bull, and Boxer (*n* = 1 each). The median age (min–max) was 86.5 months (9–171) in dogs with iPLE and 46 months (4–165) in healthy dogs; age was significantly higher in the iPLE group (*p* = 0.0002). However, within the iPLE group, no significant correlations were identified between age and serum concentrations of retinol, 25-hydroxyvitamin D, or α-tocopherol. The median body weight (min–max) of dogs with iPLE was 15.8 kg (3.4–47.5), whereas that of healthy dogs was 25.5 kg (6–55). At T0, the median (min–max) CCECAI score, and serum total protein, albumin, and cholesterol concentrations in dogs with iPLE were 8 (1–17), 4.2 g/dL (2.5–7.4), 1.8 g/dL (0.8–2.7), and 121 mg/dL (61.3–327), respectively. All dogs with iPLE underwent gastroduodenoscopy, and histopathological examination predominantly revealed lymphoplasmacytic infiltration of the intestinal mucosa. The ileum was examined in 9 dogs. The severity of duodenal lymphoplasmacytic infiltration was classified as mild in 6 dogs (10.4%), moderate in 31 dogs (53.4%), and severe in 21 dogs (36.2%). Lymphangiectasia was classified as mild in 20 dogs (34.5%), moderate in 17 dogs (29.3%), and severe in 1 dog (1.7%), whereas 20 dogs (34.5%) showed no evidence of lymphangiectasia on histopathological examination. The severity of ileal lymphoplasmacytic infiltration was classified as moderate in 7 dogs (77.8%) and severe in 2 dogs (22.2%). Lymphangiectasia was classified as mild in 3 dogs (33.3%) and moderate in 3 dogs (33.3%), whereas 3 dogs (33.3%) showed no evidence of lymphangiectasia on histopathological examination. Based on these findings, 40 dogs were assigned to group 1 and 18 dogs to group 2. Percentages may not sum to 100% because of rounding.

At T0, 28 dogs with iPLE were receiving a digestible commercial diet, 5 a nutritionally balanced homemade limited-ingredient diet formulated by a veterinary nutritionist, 12 a hydrolyzed diet, and 8 a commercial limited-ingredient diet. Five dogs were receiving mixed diet types. None of the dogs were receiving immunosuppressive therapy at T0. However, during the month preceding referral, 15 dogs had brief glucocorticoid exposure consisting of oral prednisolone administered at variable dosages ranging from 0.5 to 1 mg/kg once daily for 3–5 days. No standardized washout period was applied before referral evaluation.

### 3.2. Fat-Soluble Vitamins at T0

**Figure 1:**
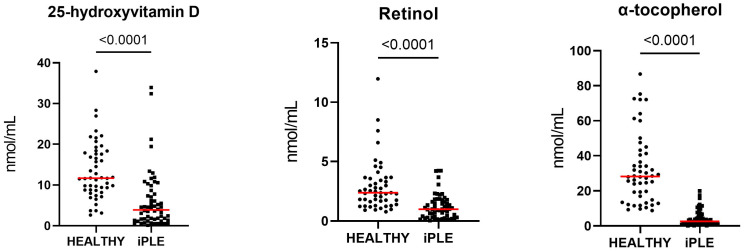
Serum concentrations of 25-hydroxyvitamin D, retinol, and α-tocopherol, expressed as nmol/mL, in healthy controls and iPLE dogs. Each dot represents an individual subject, and red horizontal lines indicate mean values in healthy controls and iPLE dogs at T0.

The median (min–max) serum retinol (vitamin A) concentration was 0.99 nmol/mL (0.04–4.2) in dogs with iPLE, compared with 2.4 nmol/mL (0.8–12) in healthy dogs. The median (min–max) serum 25-hydroxyvitamin D (vitamin D) concentration was 0.039 nmol/mL (0.0014–0.339) in dogs with iPLE and 0.117 nmol/mL (0.026–0.379) in healthy dogs. The median (min–max) serum α-tocopherol (vitamin E) concentration was 2.6 nmol/mL (0.12–19.9) in dogs with iPLE and 28.2 nmol/mL (8.8–86.7) in healthy dogs. Serum concentrations of retinol, 25-hydroxyvitamin D and α-tocopherol were significantly lower in dogs with iPLE compared with healthy dogs (all *p* < 0.0001) (Figure 1).

A weak negative correlation was observed between 25-hydroxyvitamin D concentrations and CCECAI score (*r* = −0.3; *p* = 0.02), whereas a weak positive correlation was found between 25-hydroxyvitamin D concentrations and serum albumins concentrations (*r* = 0.3; *p* = 0.02). In addition, a weak positive correlation was identified between serum α-tocopherol and retinol concentrations (*r* = 0.3; *p* = 0.02). No significant correlations were detected between α-tocopherol or retinol concentrations and CCECAI score. No other significant correlations were found between serum concentrations of 25-hydroxyvitamin D, α-tocopherol, or retinol and serum total protein, albumin, or cholesterol concentrations.

No significant differences in serum concentrations of 25-hydroxyvitamin D, α-tocopherol, or retinol were detected between dogs that had received glucocorticoids prior to enrollment and those that had not. Likewise, serum concentrations of fat-soluble vitamins did not differ significantly either between dogs assigned to histopathological groups 1 and 2 or among dogs categorized according to the diet consumed at T0 (highly digestible commercial diet, limited-ingredient homemade or commercial diets, and hydrolyzed diet).

### 3.3. Fat-Soluble Vitamins and Selected Clinicopathological Variables at T1

At T1, leftover serum samples were available for 20 dogs with iPLE. Of the 20 dogs with follow-up samples available at T1, 17 were prescribed prednisolone after T0 at variable dosages ranging from 0.5 to 1 mg/kg once daily. In addition to prednisolone, 3 dogs received chlorambucil and 1 dog received azathioprine. None of the dogs received fat-soluble vitamin supplementation. Median (min–max) CCECAI score, and serum total protein, albumin, and cholesterol concentrations in dogs with iPLE were 4 (1–14), 4.9 g/dL (2.4–7.5), 2.2 g/dL (0.9–4.6), and 121 mg/dL (60.0–395), respectively. Compared with T0, CCECAI scores and serum concentrations of total protein and albumin showed significant improvement (all *p* = 0.002). In contrast, no statistically significant difference in serum cholesterol concentrations was observed between T0 and T1. Median (min–max) serum concentrations of retinol (vitamin A), 25-hydroxyvitamin D (vitamin D) and α-tocopherol (vitamin E) in dogs with iPLE were 2.07 nmol/mL (0.06–5.25), 0.0169 nmol/mL (0.0009–0.4819), and 2.31 nmol/mL (0.34–19.82), respectively. Serum concentrations of 25-hydroxyvitamin D and α-tocopherol did not differ significantly between T0 and T1; in contrast, serum retinol concentrations were significantly higher at T1 compared with T0 (*p* = 0.01). After T0, 10 dogs were prescribed nutritionally balanced homemade low-fat diets formulated by a veterinary nutritionist, 5 dogs were prescribed hydrolyzed commercial diets, and 5 dogs were prescribed highly digestible commercial diets. At T1, serum concentrations of α-tocopherol and retinol did not differ significantly among dogs receiving highly digestible commercial diets, hydrolyzed diets, or homemade low-fat diets. However, dogs receiving homemade low-fat diets had significantly lower serum concentrations of 25-hydroxyvitamin D compared with those receiving highly digestible commercial diets (*p* = 0.02).

## 4. Discussion

The present study aimed to compare serum concentrations of vitamin A, D, and E metabolites between dogs with iPLE and healthy controls, and to explore their correlations with clinicopathological findings both at diagnosis and after one month of therapy.

Dogs with iPLE exhibited significantly reduced serum concentrations of vitamins A, D, and E metabolites compared with healthy controls, indicating a marked disruption of fat-soluble vitamin homeostasis in this condition. Fat-soluble vitamins are absorbed primarily in the mid-intestine, particularly in the jejunum and ileum, with regional differences influenced by the distribution of intestinal transporters. This process is further modulated by individual factors, including diet and genetic variability [31,32]. PLE in dogs leads to the loss of proteins and lipids into the intestinal lumen [27,33,34]. Because fat-soluble vitamins are absorbed together with dietary lipids and transported via chylomicrons and lymphatic vessels, impairment of intestinal lipid absorption and lymphatic function represents a plausible mechanism underlying the observed deficiencies [32,35].

In the present study, serum concentrations of 25-hydroxyvitamin D at T0 were significantly lower in dogs with iPLE compared with healthy controls, confirming previous reports of hypovitaminosis D in canine CE and iPLE [22,36,37]. Reduced vitamin D concentrations have also been described in dogs with exocrine pancreatic insufficiency and hepatobiliary disorders, further supporting the contribution of intestinal and hepatic dysfunction to impaired vitamin D metabolism [18,38]. In dogs with CE, decreased serum 25-hydroxyvitamin D concentrations have been associated with higher disease activity indices, greater severity of clinical signs and more severe histopathological changes [23,39]. Moreover, in severe cases of iPLE, hypovitaminosis D may result in secondary hypocalcemia and neurological complications, including seizures, and the degree of vitamin D deficiency has been shown to correlate with adverse clinical outcomes [21,40]. In the present cohort, serum 25-hydroxyvitamin D concentrations showed a weak negative correlation with CCECAI scores and a weak positive correlation with serum albumin concentrations, consistent with previous findings and suggesting that vitamin D status may reflect both inflammatory burden and protein loss [22,36]. However, the modest strength of these correlations indicates that hypovitaminosis D in iPLE is likely multifactorial rather than solely dependent on disease severity [15,41]. Proposed mechanisms include reduced intestinal absorption due to lipid malabsorption, loss of vitamin D-binding protein across the inflamed intestinal mucosa, and altered hepatic hydroxylation during systemic inflammation [22,42,43]. Because fat-soluble vitamins require carrier proteins and lipoproteins for transport in circulation, severe intestinal protein loss could theoretically influence vitamin transport dynamics in dogs with iPLE. Recent veterinary studies have suggested that dysregulation of fat-soluble vitamin homeostasis in chronic enteropathy likely reflects multifactorial mechanisms beyond intestinal malabsorption alone [25]. Circulating concentrations of specific transport proteins such as vitamin D-binding protein (VDBP) and retinol-binding protein (RBP) have not yet been extensively investigated in dogs with iPLE. Interestingly, a previous study reported similar circulating VDBP concentrations in dogs with low and normal 25(OH)D concentrations, suggesting that mechanisms other than VDBP loss may contribute to vitamin dysregulation in canine chronic enteropathies [39].

Dogs with iPLE exhibited markedly reduced serum α-tocopherol concentrations at T0 compared with healthy controls. Although data on vitamin E status in dogs with CE are limited, recent evidence suggests that alterations in vitamin E concentrations may occur as part of a broader dysregulation of fat-soluble vitamins rather than as an isolated deficiency [25,44]. Decreased serum vitamin E concentrations in dogs with iPLE are likely attributable to dietary fat malabsorption and impaired lipid transport [18,31]. In addition, increased utilization of α-tocopherol under conditions of intestinal inflammation and oxidative stress may further contribute to altered vitamin E metabolism, as described in both canine and human inflammatory intestinal diseases [11,45]. Despite sharing lipid-dependent absorption pathways with vitamins A and D, vitamin E appears disproportionately reduced in iPLE, likely because of its strong dependence on chylomicron-mediated lymphatic transport and limited extraintestinal storage, rendering it particularly vulnerable to intestinal lymphangiectasia and lipid loss [46]. In humans, chronic malabsorptive disorders have been associated with “brown bowel syndrome”, a condition linked to severe vitamin E deficiency and characterized by oxidative mitochondrial damage and lipofuscin accumulation in enteric smooth muscle, potentially exacerbating intestinal dysfunction [47]. Although this syndrome has not been described in dogs, it highlights the potential consequences of prolonged vitamin E deficiency in chronic malabsorptive states. The lack of correlation between serum α-tocopherol concentrations and CCECAI score observed in the present study suggests that vitamin E status primarily reflects altered lipid absorption rather than clinical disease severity, consistent with other previous human and veterinary studies [11,25].

Serum retinol concentrations were significantly lower at T0 in dogs with iPLE compared with healthy controls. In dogs, reduced vitamin A concentrations have been described in chronic liver disease and congenital portosystemic shunts, conditions characterized by altered hepatic storage and metabolism of retinoids [18,48]. However, the presence of biliary disease may increase serum vitamin A concentrations [18]. Although data on vitamin A status in canine CE are scarce, vitamin A is known to play a critical role in epithelial barrier integrity, immune regulation, and intestinal homeostasis [49,50]. Interestingly, Serafini et al. [25] reported higher serum retinol concentrations in dogs with CE compared with healthy controls, a finding interpreted as reflecting dysregulation of vitamin A metabolism during ongoing inflammation rather than increased intake or absorption. In contrast, dogs with iPLE in the present study exhibited reduced serum retinol concentrations at T0, likely reflecting more severe malabsorption and protein loss. Serum retinol concentrations are tightly regulated by hepatic storage and transport mechanisms and may not accurately reflect intestinal absorption, particularly in the context of chronic inflammation [46,51]. Differences in disease severity and the presence of iPLE may therefore account for the discrepant retinol profiles observed between CE and iPLE populations [25,52]. The weak positive correlation observed at T0 in the present study between serum retinol and α-tocopherol concentrations may reflect partially shared mechanisms of intestinal absorption and lipid handling, whereas differences in metabolism, storage, and regulatory pathways likely attenuate the strength of this association [46,51]. In contrast to 25-hydroxyvitamin D and α-tocopherol, serum retinol concentrations increased significantly after one month of therapy despite the absence of vitamin supplementation. This increase may reflect a rapid normalization of vitamin A metabolism following improvement of intestinal inflammation and mucosal integrity rather than a direct inverse relationship between inflammation and retinol concentrations [51]. A similar improvement in retinol status following reduction in intestinal inflammation has been reported in human patients with IBD [41]. Together, these findings suggest that vitamin A metabolism may respond more rapidly to restoration of regulatory mechanisms than other fat-soluble vitamins. Moreover, compared with α-tocopherol, retinol absorption and transport may be less dependent on intact lymphatic lipid transport, potentially explaining its earlier recovery [51]. However, the absence of significant changes in serum 25-hydroxyvitamin D and α-tocopherol concentrations after one month of therapy should be interpreted cautiously. The relatively short follow-up period may have been insufficient to detect meaningful recovery of fat-soluble vitamin status, particularly for vitamins D and E, whose restoration may require longer periods of intestinal healing, normalization of lipid absorption, and repletion of tissue stores. Longer-term prospective studies are therefore needed to better characterize the kinetics of fat-soluble vitamin recovery in dogs with iPLE.

No significant differences in serum concentrations of vitamin A, D, or E metabolites were observed between dogs that had received glucocorticoids prior to enrollment and those that had not. This finding is not surprising, as serum concentrations of fat-soluble vitamins reflect longer-term metabolic and absorptive processes, and transient glucocorticoid administration shortly before T0 may be insufficient to induce measurable changes.

No significant differences in serum concentrations of vitamin A, D, or E metabolites were observed between dogs with mild or absent lymphangiectasia and those with moderate to severe lymphangiectasia. Although intestinal lymphangiectasia is expected to disproportionately impair the absorption of α-tocopherol because of its strong dependence on chylomicron-mediated lymphatic transport, the absence of differences between groups is consistent with previous studies reporting weak associations between histopathological severity and clinical or biochemical parameters in canine CE [27,52]. The focal and patchy nature of lymphangiectasia, together with the inherent limitations of endoscopic mucosal biopsies, may lead to underestimation of the extent of lymphangiectasia [33]. Additionally, because ileal biopsies were available only in a limited number of dogs, distal small intestinal lesions potentially relevant for bile acid and fat-soluble vitamin absorption may have been underrepresented. This limitation may also have contributed to the lack of association observed between histopathological severity and vitamin concentrations. Moreover, chronic intestinal inflammation alone may be sufficient to disrupt bile acid recycling and lipid absorption, even in the absence of overt or severe lymphangiectasia, thereby blunting potential differences in fat-soluble vitamin concentrations between histological subgroups [15]. Finally, the presence of generally low vitamin concentrations across the iPLE population may have limited the ability to detect additional reductions associated with increasing lymphangiectasia severity.

Dietary type at admission was not associated with differences in fat-soluble vitamin concentrations; however, dogs fed homemade low-fat diets at follow-up had significantly lower serum 25-hydroxyvitamin D concentrations than dogs receiving highly digestible commercial diets. Low-fat diets are commonly recommended in dogs with iPLE and lymphangiectasia, but homemade formulations may be deficient in vitamin D if not adequately supplemented [53]. Additionally, dietary fat restriction may further reduce intestinal absorption of vitamin D, exacerbating pre-existing deficiencies [46]. Finally, this finding may reflect the multifactorial regulation of vitamin D metabolism, which is influenced not only by intestinal absorption but also by systemic inflammation, protein binding, and metabolic utilization during the early phase of therapeutic response [17,54]. Thus, short-term fluctuations in vitamin D concentrations may occur independently of changes in lipid absorption [17,46].

This study has several limitations. First, dogs with iPLE were significantly older than healthy control dogs. Although no significant correlations were identified between age and serum concentrations of retinol, 25-hydroxyvitamin D, or α-tocopherol, age-related differences in dietary habits, intestinal absorptive capacity, hepatic handling of vitamins, or subclinical comorbidities could still have influenced serum concentrations of fat-soluble vitamins independently of iPLE. Future studies including age-matched control populations are warranted. Second, the control population consisted of blood-donor and staff-owned dogs, which may not fully represent the general dog population. Third, follow-up serum samples were available for only a subset of cases, limiting statistical power for longitudinal analyses. Fourth, treatments administered between T0 and T1 were not standardized. Most dogs received prednisolone, some also received additional immunosuppressive drugs, and dietary management varied considerably among individuals both at diagnosis and during follow-up. Commercial diets may differ in fat-soluble vitamin content and bioavailability, whereas homemade low-fat diets, although formulated by a veterinary nutritionist, may still vary in micronutrient composition and vitamin D intake compared with commercial therapeutic diets. In addition, caloric intake and actual nutrient consumption were not quantified. Therefore, the potential influence of dietary composition, fat restriction, and vitamin intake on serum fat-soluble vitamin concentrations cannot be completely distinguished from the effects of intestinal disease and malabsorption. This limitation is particularly relevant for interpreting the lower 25-hydroxyvitamin D concentrations observed in dogs fed homemade low-fat diets, which may reflect differences in dietary vitamin D supply, formulation variability, altered intestinal absorption, or a combination of these factors. Additional methodological limitations should also be considered. Some measured concentrations were close to or below the lowest calibration standard and were estimated by extrapolation from the calibration curve. Although these values were retained for descriptive and comparative purposes, analytical accuracy may have been reduced near the lower detection limits. Furthermore, vitamin metabolites were measured in leftover serum samples stored at −80 °C. Although storage conditions were standardized and samples stored for more than 24 months were precautionarily excluded, some degree of pre-analytical variability may still have affected labile analytes, particularly retinol and α-tocopherol [29]. Retinol is especially susceptible to photodegradation and oxidative degradation, whereas α-tocopherol may also be influenced by prolonged light exposure and repeated freeze–thaw cycles. Nevertheless, all samples were processed under similar conditions and protected from light throughout the analytical workflow. Moreover, pre-analytical degradation alone is unlikely to fully explain the marked differences observed between dogs with iPLE and healthy controls. Indeed, 25-hydroxyvitamin D, which is considered more stable during storage and sample handling than retinol, was also significantly decreased in dogs with iPLE. This parallel reduction across multiple fat-soluble vitamins supports the presence of a true biological alteration in fat-soluble vitamin homeostasis rather than an exclusively analytical artifact. Finally, only serum retinol, 25-hydroxyvitamin D, and α-tocopherol concentrations were assessed. Although these analytes provide an indirect estimate of vitamin status, they may not fully reflect tissue stores or biologically active fractions. In addition, the functional consequences of altered fat-soluble vitamin homeostasis, such as oxidative stress or immune dysregulation, were not evaluated.

Despite these limitations, this study provides evidence that dogs with iPLE are at risk of concurrent alterations in multiple fat-soluble vitamins extending beyond vitamin D alone and provide a rationale for future prospective studies to define the clinical consequences of these alterations and determine the efficacy of supplementation protocols.

## 5. Conclusions

Overall, dogs with iPLE showed significantly lower serum concentrations of fat-soluble vitamins compared with healthy controls, suggesting altered fat-soluble vitamin homeostasis. Vitamin D showed modest associations with disease severity, whereas vitamins A and E appeared less directly linked to clinical indices. Notably, retinol increased after short-term therapy without supplementation, whereas no significant changes were observed for 25-hydroxyvitamin D and α-tocopherol over the one-month follow-up period. These findings support the clinical relevance of monitoring vitamin status in dogs with iPLE while highlighting the need for controlled longitudinal studies to define appropriate supplementation strategies and their impact on clinical outcomes.

## Data Availability

The summarized data supporting the findings of this study are included in this published article. Raw individual-level data are available from the corresponding author upon reasonable request.

## Abbreviations

The following abbreviations are used in this manuscript:

iPLE	inflammatory Protein-Losing Enteropathy
CE	Chronic enteropathy
